# Phenotype, outcomes and natural history of early‐stage non‐ischaemic cardiomyopathy

**DOI:** 10.1002/ejhf.3037

**Published:** 2023-10-11

**Authors:** Daniel J. Hammersley, Richard E. Jones, Ruth Owen, Lukas Mach, Amrit S. Lota, Zohya Khalique, Antonio De Marvao, Emmanuel Androulakis, Suzan Hatipoglu, Ankur Gulati, Rohin K. Reddy, Won Young Yoon, Suprateeka Talukder, Riya Shah, Resham Baruah, Kaushik Guha, Antonis Pantazis, A. John Baksi, John Gregson, John G.F. Cleland, Upasana Tayal, Dudley J. Pennell, James S. Ware, Brian P. Halliday, Sanjay K. Prasad

**Affiliations:** ^1^ National Heart and Lung Institute Imperial College London London UK; ^2^ Royal Brompton & Harefield Hospital Guy's and St Thomas' NHS Foundation Trust London UK; ^3^ Anglia Ruskin Medical School, UK Cambridge UK; ^4^ Essex Cardiothoracic Centre Basildon UK; ^5^ London School of Hygiene and Tropical Medicine London UK; ^6^ Department of Women and Children's Health King's College London London UK; ^7^ British Heart Foundation Centre of Research Excellence, School of Cardiovascular Medicine and Sciences King's College London London UK; ^8^ Lewisham and Greenwich NHS Trust London UK; ^9^ Portsmouth Hospitals NHS Trust Portsmouth UK; ^10^ British Heart Foundation Centre of Research Excellence, School of Cardiovascular and Metabolic Health University of Glasgow Glasgow UK; ^11^ MRC London Institute of Medical Sciences Imperial College London London UK

**Keywords:** Non‐ischaemic cardiomyopathy, Fibrosis, Risk stratification

## Abstract

**Aims:**

To characterize the phenotype, clinical outcomes and rate of disease progression in patients with early‐stage non‐ischaemic cardiomyopathy (early‐NICM).

**Methods and results:**

We conducted a prospective observational cohort study of patients with early‐NICM assessed by late gadolinium enhancement cardiovascular magnetic resonance (CMR). Cases were classified into the following subgroups: isolated left ventricular dilatation (early‐NICM *H−*/*D+*), non‐dilated left ventricular cardiomyopathy (early‐NICM *H+*/*D−*), or early dilated cardiomyopathy (early‐NICM *H+*/*D+*). Clinical follow‐up for major adverse cardiovascular events (MACE) included non‐fatal life‐threatening arrhythmia, unplanned cardiovascular hospitalization or cardiovascular death. A subset of patients (*n* = 119) underwent a second CMR to assess changes in cardiac structure and function. Of 254 patients with early‐NICM (median age 46 years [interquartile range 36–58], 94 [37%] women, median left ventricular ejection fraction [LVEF] 55% [52–59]), myocardial fibrosis was present in 65 (26%). There was no difference in the prevalence of fibrosis between subgroups (*p* = 0.90), however fibrosis mass was lowest in early‐NICM *H−*/*D+*, higher in early‐NICM *H+*/*D−* and highest in early‐NICM *H+*/*D+* (*p* = 0.03). Over a median follow‐up of 7.9 (5.5–10.0) years, 28 patients (11%) experienced MACE. Non‐sustained ventricular tachycardia (hazard ratio [HR] 5.1, 95% confidence interval [CI] 2.36–11.00, *p* < 0.001), myocardial fibrosis (HR 3.77, 95% CI 1.73–8.20, *p* < 0.001) and diabetes mellitus (HR 5.12, 95% CI 1.73–15.18, *p* = 0.003) were associated with MACE in a multivariable model. Only 8% of patients progressed from early‐NICM to dilated cardiomyopathy with LVEF <50% over a median of 16 (11–34) months.

**Conclusion:**

Early‐NICM is not benign. Fibrosis develops early in the phenotypic course. In‐depth characterization enhances risk stratification and might aid clinical management.

## Graphical abstract

Patients with early‐stage non‐ischaemic cardiomyopathy (early‐NICM) were characterized with late gadolinium enhancement (LGE) cardiovascular magnetic resonance. Three subgroups were included: isolated left ventricular (LV) dilatation, non‐dilated LV cardiomyopathy and early dilated cardiomyopathy (DCM). Myocardial fibrosis was identified in a quarter of patients and was equally ubiquitous across subgroups. Fibrosis mass was lowest in isolated LV dilatation, higher in non‐dilated LV cardiomyopathy and highest in early DCM. During long‐term follow‐up, 11% of patients experienced major adverse cardiovascular events (MACE). Fibrosis presence, non‐sustained ventricular tachycardia and diabetes mellitus were independently associated with MACE, whereas a family history of dilated cardiomyopathy was associated with lower incidence of MACE.

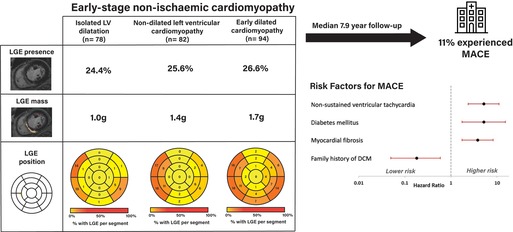

## Introduction

With the expansion of family screening, genetic testing and advanced cardiovascular imaging, more patients are being diagnosed with early‐stage non‐ischaemic cardiomyopathy (early‐NICM), usually before the onset of symptoms. Mild abnormalities of cardiac structure and function may, or may not, fulfill the full diagnostic criteria for non‐ischaemic dilated cardiomyopathy (DCM), depending on whether left ventricular (LV) dilatation and systolic dysfunction exist together or in isolation.^1^ When present in isolation, intermediate phenotypes may exist, namely isolated LV dilatation or non‐dilated LV cardiomyopathy.^2^ Rare variants in DCM genes are similarly prevalent amongst patients with non‐dilated LV cardiomyopathy and DCM, supporting the notion that these phenotypes reflect a spectrum of the same disease.^3^ Observational studies of patients with early‐NICM are lacking. There are limited data characterizing phenotype, disease progression and stratifying risk.^2^ Accordingly, there remains uncertainty regarding optimal management and limited consensus in guidelines.^4^, ^5^

Many patients with new‐onset symptomatic DCM respond well to contemporary heart failure (HF) therapies, resulting in high rates of LV reverse remodelling.^6^, ^7^ Whether pre‐emptively initiating these medications in the pre‐clinical phase attenuates disease progression, reduces transition to symptomatic HF and improves outcomes requires further evaluation. Amidst aspiration to transition from treatment to prevention of HF, patients with early disease represent an attractive group for targeted therapy. The molecular mechanisms driving early disease across the spectrum of DCM are unknown.^8^ Before trials evaluating the efficacy of early therapy can be designed, it is important to understand more about the characteristics, natural history and risk of early‐NICM. In this study, we characterize the phenotype of early‐NICM using cardiovascular magnetic resonance (CMR), evaluate the risk of adverse cardiovascular outcomes and assess the rate of phenotypic progression.

## Methods

### Study population

Consecutive patients referred for a CMR between 2009 and 2017 from our clinical service and network of surrounding hospitals were prospectively enrolled into the Royal Brompton Hospital Cardiovascular Research Centre (RBHCRC) Biobank. The study complied with the Declaration of Helsinki and was approved by the National Research Ethics Service (South Central Hampshire B, Reference 19/SC/0257). All participants provided written informed consent. The early‐NICM cohort comprised three patient subgroups classified on the basis of increased indexed LV end‐diastolic volume (LVEDVi) and/or reduced LV ejection fraction (LVEF) measured by CMR: (i) isolated LV dilatation (early‐NICM *H−*/*D+*_,_ denoting early‐NICM without LV hypokinesia but with LV dilatation compared to age‐ and sex‐specific reference nomograms for LVEF and LVEDVi, respectively);^9^ (ii) non‐dilated LV cardiomyopathy (early‐NICM *H+*/*D−*), comprising patients with reduced LVEF with normal LVEDVi; and (iii) early DCM (early‐NICM *H+*/*D+*), comprising patients with LVEF ≥50% but less than age‐ and sex‐specific reference nomogram value and increased LVEDVi (online supplementary *Figure* *S1*). To ensure patients with later‐stage DCM were not included, those with reverse remodelled DCM (prior LVEF <40%, improved to ≥50%), previous HF admissions or New York Heart Association (NYHA) class >II were excluded. Additional exclusion criteria were ischaemic heart disease (IHD), defined as stenosis of >50% in a major epicardial coronary artery, inducible ischaemia on functional testing, subendocardial late gadolinium enhancement (LGE) on CMR indicating prior infarction, or prior coronary revascularization; further exclusion criteria were abnormal loading conditions (uncontrolled hypertension or severe primary valve disease), congenital heart disease, active myocarditis, or an alternative cardiomyopathy. Subjects considered by expert consensus opinion from within our department to have abnormal LVEDVi/LVEF due to athletic remodelling were excluded.^10^ Patients with prior myocarditis but without active inflammation were included where cases met the pre‐specified LVEDVi/LVEF criteria, in line with recent work illustrating a significant phenotypic and genetic overlap with NICM.^11^

A separate cohort of patients with later‐stage DCM enrolled in the RBHCRC Biobank were included (DCM cohort) for comparison. Inclusion criteria for the DCM group were confirmed DCM with LVEF <50% and increased LVEDVi, in the absence of significant IHD, adverse loading conditions, an alternative cardiomyopathy or congenital heart disease. Baseline characteristics included prior non‐sustained ventricular tachycardia (VT), which was captured on Holter monitoring performed as part of routine clinical evaluation prior to enrolment.

### Cardiovascular magnetic resonance

All patients underwent a CMR scan at 1.5 Tesla (Sonata/Avanto, Siemens, Erlangen, Germany). Breath‐hold steady‐state free precession sequences were performed to produce long‐ and short‐axis cine images. Gadopentetate dimeglumine or gadobutrol (0.1 mmol/kg) was injected intravenously and an inversion recovery gradient echo sequence undertaken to acquire LGE images at 10 min. Left and right ventricular volumes and LV mass were measured using CMRtools (Cardiovascular Imaging Solutions, London, UK). LGE presence was assessed by two independent expert CMR readers, with a third adjudicating cases of disagreement. LGE was defined as an area of enhancement within the intramyocardial and/or subepicardial layers and considered present when seen in both long‐ and short‐axis planes, in two orthogonal views, extending beyond the LV/right ventricle (RV) insertion points. LV/RV insertion point enhancement alone was not classified as LGE. LGE position was classified as septal/free‐wall and using the American Heart Association 17‐segment model. LGE pattern was classified as mid‐wall/subepicardial. LGE was quantified on CVI42 (Circle Cardiovascular Imaging Inc, Calgary, Canada) using 5‐standard deviation (5SD) signal threshold versus reference myocardium method by an expert operator blinded to phenotypic subgroup classification and outcomes. LGE quantification is presented as both absolute mass (g) and relative mass (% myocardial mass).

### Follow‐up and endpoints

Patients were followed up using postal questionnaires and from primary care and hospital records. Follow‐up duration was measured from CMR date and truncated at 12 years. Patients were censored at first event. The process for identifying events was identical between early‐NICM and DCM cohorts. Events were adjudicated by a panel of experienced cardiologists using medical information, death certificates, autopsy reports and ICD reports. The primary endpoint was major adverse cardiovascular events (MACE), defined in this study as a composite of cardiovascular mortality, non‐fatal life‐threatening arrhythmic events (aborted sudden cardiac death [aSCD] or haemodynamically unstable sustained VT) or unplanned cardiovascular hospitalization. Secondary endpoints were (i) life‐threatening arrhythmia [LTA] (sudden cardiac death [SCD], aSCD or haemodynamically unstable VT); and (ii) major HF events (HF death, heart transplant, left ventricular assist device (LVAD) implantation, or HF hospitalization).^12^

### Phenotypic progression assessment

To evaluate phenotypic progression in patients with early‐NICM, data on a subset of patients that had at least one further CMR performed on clinical grounds after enrolment were obtained. If the phenotype had progressed so that both LVEDVi was greater than age‐ and sex‐specific reference nomogram values and LVEF was <50%, this was regarded as progression from early‐NICM to DCM.

### Statistical analysis

Patient characteristics were presented as frequency (%) for categorical variables and median (interquartile range) for continuous variables. Categorical variables were compared using χ^2^ test or Fisher's exact test. Continuous variables were compared using 2‐sample *t*‐test, ANOVA, Mann–Whitney U test or Kruskal–Wallis. Ordinal values were compared using test for trend. Cumulative incidence curves were estimated using Kaplan–Meier method and compared using the log‐rank test. The association between patient characteristics and the primary endpoint was examined using univariable and multivariable Cox proportional hazard modelling. Multivariable models were built using forward stepwise selection method of candidate variables with an entry criterion of *p* < 0.05 (online supplementary *Table* *S4*); this approach was selected due to the absence of established risk factors for MACE in this population. A 2‐sided *p* < 0.05 was considered statistically significant. Statistical analyses were performed using Stata v17 (StatCorp, College Station, TX, USA) and SPSS v27 (IBM, Armonk, NY, USA).

## Results

### Cohort

Of 1282 patients assessed for eligibility, the final early‐NICM cohort consisted of 254 patients (78 early‐NICM *H−*/*D+*, 82 early‐NICM *H+*/*D−*, 94 early‐NICM *H+*/*D+*) (online supplementary *Figure* *S2*). The most common indication for CMR was to investigate mild LV dysfunction or dilatation identified on echocardiography (87 patients [34%]), followed by cardiomyopathy family screening (76 [30%]) and arrhythmia (49 [19%] including 40 for ventricular ectopy/arrhythmia, seven for atrial arrhythmia and two for ventricular ectopy/arrhythmia with atrial arrhythmia). The median time interval from initial diagnosis to CMR was 0 months (0–0.8 months). The median age of all patients with early‐NICM was 47 (36–58) years (*Table* *1*). Patients with early‐NICM *H+*/*D−* had higher resting heart rate than patients with early‐NICM *H+*/*D+* and early‐NICM *H−*/*D+* (*p* < 0.001), and a higher proportion of patients with early‐NICM *H+*/*D−* had a history of atrial fibrillation/flutter than early‐NICM *H+*/*D+* and early‐NICM *H−*/*D+* (*p* < 0.001). Non‐sustained VT had been identified in 21 patients (12%) and was most prevalent in early‐NICM *H+*/*D+* (*p* = 0.01). A higher proportion of patients with early‐NICM *H+*/*D−* and early‐NICM *H+*/*D+* were treated with angiotensin‐converting enzyme inhibitors/angiotensin receptor blockers (ACEi/ARB) and beta‐blockers than those with early‐NICM *H−*/*D+* (both *p* < 0.001).

**Table 1:** Baseline characteristics of patients with early‐stage non‐ischaemic cardiomyopathy

	All early‐NICM patients (*n* = 254)	Early‐NICM subgroups			*p*‐value
		Early‐NICM *H−*/*D+* (*n* = 78)	Early‐NICM *H+*/*D−* (*n* = 82)	Early‐NICM *H*+/*D*+ (*n* = 94)	
Age, years	47 (36–58)	45 (31–54)	45 (36–58)	52 (41–63)	<0.001
Women	94/254 (37)	27/78 (35)	30/82 (37)	37/94 (39)	0.81
Caucasian	234/254 (92)	76/78 (97)	75/82 (91)	83/94 (88)	0.08
Heart rate, bpm	70 (60–80)	65 (57–75)	75 (65–87)	67 (60–76)	<0.001
Systolic blood pressure, mmHg	124 (112–138)	125 (113–138)	121 (110–132)	128 (113–139)	0.28
Diastolic blood pressure, mmHg	73 (65–82)	71 (63–78)	73 (64–83)	74 (68–82)	0.12
Body mass index, kg/m^2^	26.0 (22.7–29.5)	25.9 (22.8–29.4)	26.7 (22.7–30.5)	25.3 (22.7–28.3)	0.17
Diabetes mellitus	7/254 (3)	1/78 (1)	3/82 (4)	3/94 (3)	0.62
Hypertension	60/254 (24)	15/78 (19)	18/82 (22)	27/94 (29)	0.31
Current smoker	33/252 (13)	12/78 (15)	12/82 (15)	9/92 (10)	0.49
History of excess alcohol	22/231 (10)	2/69 (3)	10/77 (13)	10/85 (12)	0.08
History of chemotherapy	6/203 (3)	2/57 (4)	2/67 (3)	2/79 (3)	1.00
Peripartum presentation	2/235 (1)	1/76 (1)	1/74 (1)	0/85 (0)	0.54
History of inherited muscular disease	1/207 (1)	0/59 (0)	1/69 (1)	0/79 (0)	0.62
Family history of DCM	85/211 (40)	32/61 (52)	36/73 (49)	17/77 (22)	<0.001
Family history of SCD	45/202 (22)	14/57 (25)	16/70 (23)	15/75 (20)	0.81
Atrial fibrillation/flutter	44/228 (19)	6/70 (9)	26/75 (35)	12/83 (15)	<0.001
Left bundle branch block	29/254 (11)	4/78 (5)	6/82 (7)	19/94 (20)	0.003
Non‐sustained VT	21/176 (12)	5/57 (9)	3/59 (5)	13/60 (22)	0.01
NYHA class					<0.001
I	181/254 (71)	69/78 (88)	50/82 (61)	62/94 (66)	
II	73/254 (29)	9/78 (12)	32/82 (39)	32/94 (34)	
CMR indication					0.002
Characterization of LV dysfunction/dilatation	87/254 (34)	15/78 (19)	34/82 (42)	38/94 (40)	
Arrhythmia	49/254 (19)	17/78 (22)	14/82 (17)	18/94 (19)	
Family screen	76/254 (30)	35/78 (45)	24/82 (29)	17/94 (18)	
Other	42/254 (17)	11/78 (14)	10/82 (12)	21/94 (22)	
ACEi or ARB	132/253 (52)	26/77 (34)	46/82 (56)	60/94 (64)	<0.001
Beta‐blocker	97/253 (38)	12/77 (16)	38/82 (46)	47/94 (50)	<0.001
Mineralocorticoid receptor antagonist	20/253 (8)	4/77 (5)	7/82 (9)	9/94 (10)	0.55

Data presented as median (interquartile range) or *n/N* (%).

ACEi, angiotensin‐converting enzyme inhibitor; ARB, angiotensin II receptor blocker; CMR, cardiovascular magnetic resonance; DCM, dilated cardiomyopathy; early‐NICM *H*−/*D*+, isolated left ventricular dilatation; early‐NICM *H*+/*D*−, non‐dilated left ventricular cardiomyopathy; early‐NICM *H*+/*D*+, early DCM; early‐NICM, early non‐ischaemic cardiomyopathy; LV, left ventricular; NYHA, New York Heart Association; SCD, sudden cardiac death; VT, ventricular tachycardia.

The DCM comparator cohort consisted of 540 patients. Compared to the DCM cohort, patients with early‐NICM were younger, had lower heart rate and higher systolic blood pressure (online supplementary *Table* *S1*). There was a lower burden of comorbidity amongst patients with early‐NICM than DCM, including lower body mass index, and lower prevalence of diabetes and hypertension.

### Phenotype of early‐stage non‐ischaemic cardiomyopathy

**Figure 1:**
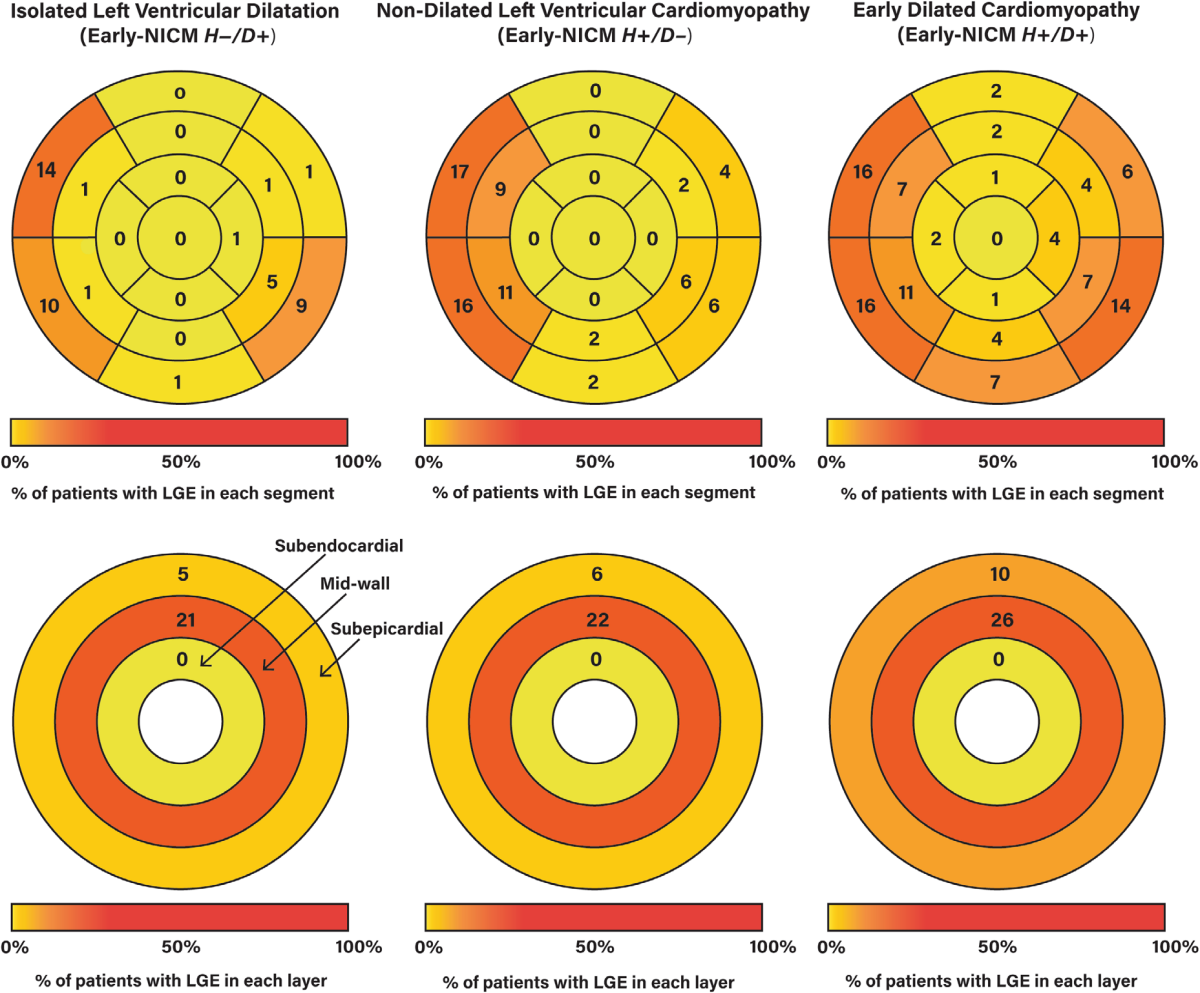
Late gadolinium enhancement (LGE) position and pattern in patients with early‐stage non‐ischaemic cardiomyopathy (NICM). American Heart Association 17‐segment model is used to represent LGE position. Three myocardial layers (subepicardial, mid‐wall and subendocardial) are used to represent late gadolinium pattern. In patients with isolated left ventricular dilatation, fibrosis was mostly identified at the basal level_,_ whereas fibrosis was more frequently found at basal and mid‐level in non‐dilated left ventricular cardiomyopathy and early dilated cardiomyopathy. The proportion of patients with fibrosis in each myocardial layer was similar between subgroups. Early‐NICM *H−*/*D+*, isolated left ventricular dilatation; early‐NICM *H+*/*D−*, non‐dilated left ventricular cardiomyopathy; early‐NICM *H+*/*D+*, early dilated cardiomyopathy.

The median LVEF of all patients with early‐NICM was 55% (52–59), including early‐NICM *H−*/*D+* subgroup median LVEF 61% (59–63), early‐NICM *H+*/*D−* subgroup median LVEF 52% (49–55), and early‐NICM *H+*/*D+* subgroup median LVEF 54% (52–56). While those with early‐NICM *H+*/*D+* had the highest indexed LV mass (*p* = 0.002), no difference was seen in LV wall thickness between subgroups indicating greater eccentric LV remodelling in early‐NICM *H+*/*D+*. Myocardial fibrosis was found in 65 (26%) patients with early‐NICM. Importantly, there was no difference in the prevalence of fibrosis between subgroups (19/78 [24%] amongst early‐NICM *H−*/*D+*; 21/82 [26%] amongst early‐NICM *H+*/*D−*; 25/94 [27%] amongst early‐NICM *H+*/*D+*, *p* = 0.90). There was also no difference between early‐NICM subgroups in either the position of fibrosis (septal/free‐wall) or pattern (mid‐wall/subepicardial). Of those with fibrosis, absolute fibrosis mass was slightly lower in the early‐NICM *H−*/*D+* subgroup (1.0 g [0.5–2.0]) compared to the early‐NICM *H+*/*D−* subgroup (1.4 g [1.1–2.2]), and early‐NICM *H+*/*D+* subgroup (1.7 g [1.2–4.8]) (*p* = 0.03). The difference in fibrosis mass between subgroups remained after indexing to LV mass (relative fibrosis mass) (*p* = 0.04) (*Table* *2*). American Heart Association models of scar position demonstrated a predominantly basal fibrosis distribution in early‐NICM *H−*/*D+*_,_ whereas those with early‐NICM *H+*/*D−* and early‐NICM *H+*/*D+* had a higher proportion with both basal and mid‐cavity fibrosis. The proportion of patients with fibrosis in each myocardial layer was similar among subgroups (*Figure* *1*).

**Table 2:** Phenotype of patients with early‐stage non‐ischaemic cardiomyopathy

	All early‐NICM patients				*p*‐value
	All early‐NICM patients (*n* = 254)	Early‐NICM *H−*/*D+* (*n* = 78)	Early‐NICM *H+*/*D−* (*n* = 82)	Early‐NICM *H+*/*D+* (*n* = 94)	
LVEDVi, ml/m^2^	101 (90–108)	105 (101–111)	85 (77–91)	107 (100–117)	<0.001
LVESVi, ml/m^2^	44 (40–50)	41 (37–44)	41 (37–44)	50 (45–56)	<0.001
LVEF, %	55 (52–59)	61 (59–63)	52 (49–55)	54 (52–56)	<0.001
Indexed LV mass, g/m^2^	74 (64–83)	73 (64–83)	69 (59–77)	77 (68–88)	0.002
RVEDVi, ml/m^2^	91 (78–106)	104 (91–116)	78 (69–89)	93 (80–106)	<0.001
RVESVi, ml/m^2^	39 (31–47)	42 (32–51)	38 (31–44)	38 (31–49)	0.04
RVEF, %	57 (52–63)	59 (55–65)	53 (47–58)	58 (54–63)	<0.001
LAVi, ml/m^2^	51 (42–61)	53 (47–62)	42 (35–51)	56 (46–66)	<0.001
Maximum LV wall thickness, mm	10.0 (8.0–11.0)	9.0 (8.0–11.0)	10.5 (8.0–12.0)	10.0 (8.0–11.0)	0.09
Mean septal thickness, mm	7.5 (6.0–9.0)	7.0 (6.0–9.0)	8.5 (6.5–9.0)	7.5 (6.0–9.0)	0.57
Mean lateral wall thickness, mm	5.5 (4.0–6.5)	5.0 (4.0–6.0)	5.5 (5.0–7.0)	5.5 (4.0–6.5)	0.38
Myocardial fibrosis present (%)	65/254 (26)	19/78 (24)	21/82 (26)	25/94 (27)	0.90
Fibrosis position (% of patients)					
Septal	45/254 (18)	14/78 (18)	15/82 (18)	16/94 (17)	0.97
Free‐wall	34/254 (13)	8/78 (10)	9/82 (11)	17/94 (18)	0.24
Fibrosis pattern (% of patients)					
Mid‐wall	59/254 (23)	17/78 (22)	18/82 (22)	24/94 (26)	0.80
Subepicardial	18/254 (7)	4/78 (5)	5/82 (6)	9/94 (10)	0.48

	Early‐NICM patients with myocardial fibrosis				
	Early‐NICM patients with fibrosis (*n* = 65)	Early‐NICM *H−*/*D+* (*n* = 19)	Early‐NICM *H+*/*D−* (*n* = 21)	Early‐NICM *H+*/*D+* (*n* = 25)	
Fibrosis mass (5SD method)					
Absolute mass, g	1.6 (0.9–2.7)	1.0 (0.5–2.0)	1.4 (1.1–2.2)	1.7 (1.2–4.8)	0.03
Relative mass, %	1.2 (0.7–2.1)	0.8 (0.4–1.7)	1.1 (0.9–2.0)	1.5 (0.9–3.5)	0.04

Data presented as median (interquartile range) or *n/N* (%).

5SD, 5‐standard deviation; early‐NICM *H−*/*D+*, isolated left ventricular dilatation; early‐NICM *H+*/*D−*, non‐dilated left ventricular cardiomyopathy; early‐NICM *H+*/*D+*, early dilated cardiomyopathy; early‐NICM, early non‐ischemic cardiomyopathy; LAVi, left atrial volume index; LV, left ventricular; LVEDVi, left ventricular end‐diastolic volume index; LVEF, left ventricular ejection fraction; LVESVi, left ventricular end‐systolic volume index; RVEDVi, right ventricular end‐diastolic volume index; RVEF, right ventricular ejection fraction; RVESVi, right ventricular end‐systolic volume index.

When compared to patients with DCM, fewer patients with early‐NICM had myocardial fibrosis (65/254 [26%] vs. 207/540 [38%], *p* < 0.001), including a lower proportion with fibrosis involving the mid‐wall (*p* < 0.001) and septum (*p* < 0.001). Patients with early‐NICM and fibrosis had lower absolute fibrosis mass compared to DCM with fibrosis (1.6 g [0.9–2.7] vs. 2.2 g [1.3–3.6], *p* = 0.007); but did not have lower relative fibrosis mass (1.2% [0.7–2.1%] vs. 1.5% [0.8–2.4%]. *p* = 0.52), due to the effect of indexing to larger LV mass values in the DCM group (online supplementary *Table* *S2*).

### Clinical outcomes in early‐stage non‐ischaemic cardiomyopathy

#### Major adverse cardiovascular events

**Figure 2:**
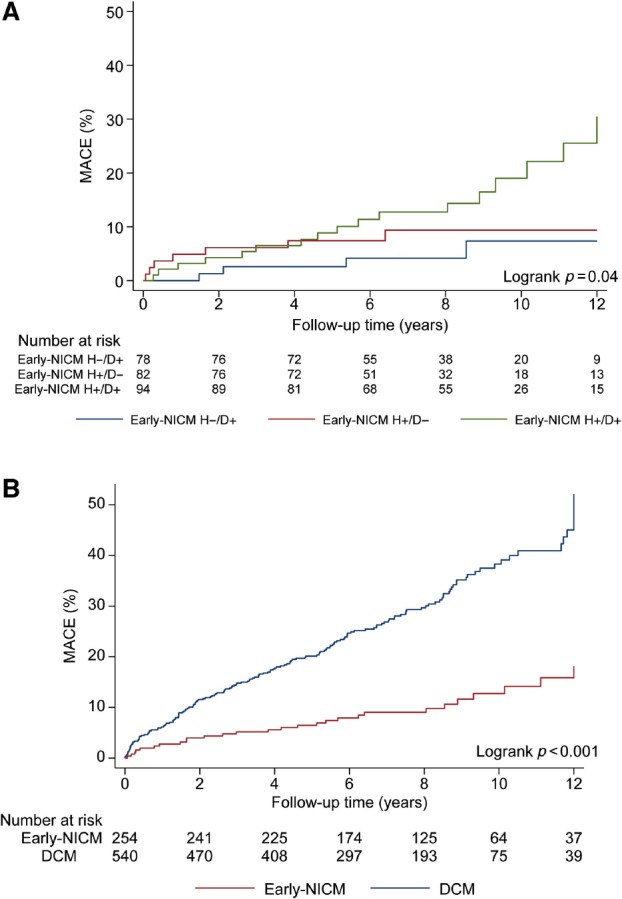
Cumulative incidence of major adverse cardiovascular events (MACE) stratified by (*A*) early non‐ischaemic cardiomyopathy (NICM) subgroups; and (*B*) early‐NICM versus dilated cardiomyopathy (DCM) with left ventricular ejection fraction <50%. Results are suggestive of a higher cumulative incidence of MACE in patients with early‐DCM (early‐NICM *H+*/*D+*) compared to patients with non‐dilated left ventricular cardiomyopathy (early‐NICM *H+*/*D−*) or isolated left ventricular dilatation (Early‐NICM *H−*/*D+*) (*A*). Patients with DCM with left ventricular ejection fraction <50% had a higher cumulative incidence of MACE than patients with early NICM (*B*). Early‐NICM *H−*/*D+*, isolated left ventricular dilatation; early‐NICM *H+*/*D−*, non‐dilated left ventricular cardiomyopathy; early‐NICM *H+*/*D+*, early dilated cardiomyopathy.

Over a median follow‐up of 7.9 (5.5–10.0) years, 28/254 (11%) patients with early‐NICM experienced MACE. This was adjudicated as cardiovascular death in two patients (both SCD), non‐fatal LTA in eight patients (seven aSCDs and one haemodynamically unstable sustained VT), and unplanned cardiovascular hospitalization in 18 patients (seven with HF, five with atrial arrhythmia, two due to bradyarrhythmia, one with cardiac syncope that preceded an aSCD event, one with a cardiovascular procedural complication, one with symptomatic secondary mitral regurgitation due to progressive LV dilatation and systolic impairment, and one with an acute coronary syndrome 7 years after enrolment and 8 years after invasive angiography showing unobstructed coronary arteries). The median time interval from CMR to MACE was 4.0 (1.1–7.6) years amongst those who met the primary endpoint. Our results suggested a higher cumulative incidence of MACE in the early‐NICM *H+*/*D+* subgroup compared to early‐NICM *H+*/*D*− and early‐NICM *H*−/*D+* (log‐rank *p* = 0.04; *Figure* *2A*), although notably separation of cumulative incidence curves was only observed after 7 years, which was approximately the median duration of follow‐up in the cohort. Patients with early‐NICM collectively had a lower cumulative incidence of MACE than patients with DCM (log‐rank *p* < 0.001; *Figure* *2B*). Patients with early‐NICM that experienced MACE were older, had a higher body mass index, a lower proportion were Caucasian and a higher proportion had diabetes mellitus, prior non‐sustained VT and were treated with ACEi/ARB, beta‐blockers or mineralocorticoid receptor antagonists compared to those with early‐NICM that did not experience MACE. A lower proportion of patients with early‐NICM that experienced MACE had a family history of DCM than those that did not experience MACE. There was no difference in baseline LVEF between patients with early‐NICM that did and did not experience MACE, although those that did experience MACE had higher LVESVi, higher left atrial volume index values and a higher proportion had myocardial fibrosis (online supplementary *Table* *S3*). The variables independently associated with higher incidence of MACE on multivariable analysis in patients with early‐NICM were non‐sustained VT (hazard ratio [HR] 5.10, 95% confidence interval [CI] 2.36–11.00, *p* < 0.001), myocardial fibrosis presence (HR 3.77, 95% CI 1.73–8.20, *p* < 0.001) and diabetes (HR 5.12, 95% CI 1.73–15.18, *p* = 0.003). A family history of DCM was independently associated with lower incidence of MACE in early‐NICM (HR 0.18, 95% CI 0.05–0.59, *p* = 0.005) (online supplementary *Table* *S4*).

#### Life‐threatening arrhythmic events

Of the 254 patients with early‐NICM, 11 (4%) experienced LTA during follow‐up. These included eight patients with aSCD events (four with resuscitated cardiac arrests in patients without implantable cardioverter‐defibrillators [ICDs], four with appropriate ICD shocks from ICDs implanted during follow‐up), one patient with haemodynamically unstable VT causing syncope detected on ICD and treated with anti‐tachycardia pacing, and two patients adjudicated to have died from SCD. One patient who died suddenly had ventricular fibrillation (VF) detected on implantable loop recorder interrogation, while the other had an out‐of‐hospital VF arrest with unsuccessful resuscitation. Neither had LVEF <35% or an ICD. Of the 11 patients that experienced LTA, only one had a LVEF <35% documented prior to the LTA (LVEF 30%). No difference was found in the cumulative incidence of LTA between early‐NICM subgroups (log‐rank *p* = 0.95); patients with early‐NICM had a lower cumulative incidence of life‐threatening arrhythmic events than patients with DCM (log‐rank *p* = 0.03) (online supplementary *Figure* *S3*). In total, 26/254 (10%) patients with early‐NICM underwent ICD/cardiac resynchronization therapy‐defibrillator (CRT‐D) implantation during follow‐up, of which five were for secondary prevention (four following resuscitated cardiac arrest occurring during follow‐up, one following resuscitated cardiac arrest occurring prior to enrolment, with device implantation after enrolment). The remaining 21 had ICD/CRT‐D implants for primary prevention, including eight patients in whom the indication related to subsequent severe LV systolic impairment (one of whom had had a permanent pacemaker implanted for complete heart block and underwent CRT‐D upgrade), eight patients in whom the indication related to non‐sustained VT (two of whom additionally reported syncope, one of whom additionally had significant sinus pauses), a further four patients in whom lamin A/C variants were identified on genetic testing performed on clinical grounds (two of whom additionally had non‐sustained VT, one of whom additionally had conduction disease), and one patient who underwent CRT‐D implant due to syncope, left bundle branch block and moderate LV systolic impairment. The indication for ICD implantation in the four early‐NICM patients who had appropriate ICD shocks was NSVT on cardiac monitoring or exercise testing, and in one case non‐sustained VT and bradycardia.

#### Heart failure events

Amongst the 254 patients with early‐NICM, nine (4%) met the HF endpoint during follow‐up, all from HF hospitalizations. Of these, one patient went on to cardiac transplantation, and none had LVADs implanted or died from HF. No difference was found in the cumulative incidence of major HF events between early‐NICM subgroups (log‐rank *p* = 0.22). Patients with early‐NICM collectively had a lower cumulative incidence of HF events than patients with DCM (log‐rank *p* < 0.001) (online supplementary *Figure* *S4*).

#### Relative burden of disease in early‐stage non‐ischaemic cardiomyopathy

While patients with early‐NICM had a lower incidence of both LTA and HF events than patients with DCM, they were proportionally less susceptible to HF events than to LTA. The rate ratio between DCM and early‐NICM cohorts for LTA was 1.87 (95% CI 0.94–4.05), but for major HF events was 6.54 (95% CI 3.32–14.71) (online supplementary *Table* *S5*).

### Phenotypic progression of early‐stage non‐ischaemic cardiomyopathy

Of the 254 patients with early‐NICM, 119 (47%) underwent a further CMR at our centre (36 early‐NICM *H−*/*D+*, 38 early‐NICM *H+*/*D−* and 45 early‐NICM *H+*/*D+*). The median time interval between CMRs was 16 months (11–34). In total, 9/119 (8%) patients progressed from early‐NICM to DCM on the second CMR. Compared to the patients that did not progress, those that did progress to DCM had a higher baseline heart rate (89 [69–100] vs. 70 [61–77] bpm, *p* < 0.001) and a higher proportion were smokers, consumed excess alcohol, had left bundle branch block and were prescribed beta‐blockers (online supplementary *Table* *S6*). Those that progressed to DCM had higher baseline LVESVi (50 [43–52] vs. 43 [38–47] ml/m^2^, *p* = 0.04) and lower LVEF (54% [52–55%] vs. 57% [54–59%], *p* = 0.02) compared to those that did not progress (online supplementary *Table* *S7*). Of the nine patients from this subset that progressed to DCM, two had LVEF <35% at follow‐up CMR and went on to have devices implanted, the remaining seven had LVEF values between 35–50% and did not undergo device implantation. None of these nine patients experienced MACE during follow‐up.

## Discussion

### Myocardial fibrosis occurs early in the phenotypic course of non‐ischaemic cardiomyopathy

To date, the time point at which myocardial fibrosis formation occurs and is detectable on CMR has not been reported in patients with early‐NICM. We demonstrate that mid‐wall/subepicardial fibrosis was present in a quarter of patients with early‐NICM and was similarly ubiquitous in each early‐NICM subgroup (*Graphical Abstract*). This finding indicates that myocardial fibrosis deposition occurs at an early point in the phenotypic course of NICM, often preceding significant adverse cardiac remodelling and symptom onset. Our findings are consistent with a previous biopsy study of people with isolated LV dilatation who were family members of patients with DCM. A quarter had histological evidence of myocyte pleomorphism and interstitial fibrosis and immunohistochemistry revealed similar inflammatory cellular infiltrates between patients with DCM and their relatives with isolated LV dilatation.^13^ It has been postulated that myocardial fibrosis may be epiphenomenon in DCM, occurring as a by‐product of reparative processes activated by cardiomyocyte injury caused by higher LV wall stress.^14^ Our results do not support this theory and instead suggest that myocardial fibrosis is a distinct process that is physiologically uncoupled from adverse remodelling during the early stages of disease. A similar phenomenon has been observed in subjects with pathogenic sarcomeric mutations associated with hypertrophic cardiomyopathy, in whom elevated serological markers of fibrosis precede the overt LV hypertrophy that defines the hypertrophic cardiomyopathy phenotype.^15^

### Fibrosis mass differs between early‐stage non‐ischaemic cardiomyopathy subgroups

The finding of a gradient in fibrosis mass across early‐NICM subgroups with even more fibrosis amongst patients with DCM suggests that it may be an integral part of disease progression rather than a static phenomenon from a single insult. These observations support work from Mandawat *et al*.,^16^ who reported progression of fibrosis in 18% of DCM cases over a median of 1.5 years, frequently occurring in tandem with adverse ventricular remodelling, and associating with HF events and death.

### Incidence of major adverse cardiovascular events and proportional risk of arrhythmia in early‐stage non‐ischaemic cardiomyopathy

An important finding from this study was that 11% of patients with early‐NICM experienced MACE over long‐term follow‐up. These findings do not support the notion that early‐NICM is a benign condition, and instead indicate that risk stratification and surveillance are important. Although patients with early‐NICM had a much lower event rate than those with DCM, they had a proportionally higher burden of arrhythmic events compared to HF events. This observation may be explained by the major arrhythmic substrate being fibrosis, which was a common feature of early‐NICM, whereas HF events generally occur in the setting of significant LV systolic dysfunction. We suspect there may be some patients with early‐NICM who are more susceptible to arrhythmias, such as those with variants in filamin C, which is associated with mild LV systolic dysfunction but increased risk of LTA.^17^, ^18^ The higher proportional risk of LTA compared to HF in early‐NICM, coupled with the youth of this population, and their low risk of dying from HF or comorbid disease, raises the question of whether they might get a substantial long‐term survival benefit from a primary prevention ICD, as they are unlikely to die from competing non‐sudden causes. Importantly, few patients who experienced LTA had a decrement in LVEF to <35% prior to their event and most would not have met guideline eligibility criteria for a primary prevention ICD, suggesting that better methods of risk stratification than LVEF are required. This study suggests that fibrosis, non‐sustained VT and diabetes might be useful for risk stratification. These findings have implications for clinical practice, supporting investigation with CMR and rhythm monitoring and lifestyle management for prevention of diabetes. A family history of DCM was associated with lower incidence of MACE, which could be explained by earlier disease detection and closer monitoring due to family screening.

### The rate of phenotypic progression from early‐stage non‐ischaemic cardiomyopathy is low over short‐term follow‐up

The proportion of patients with early‐NICM progressing to DCM over short‐term follow‐up was low, in keeping with findings by Hazebroek *et al*.,^3^ who reported a 2% annual progression rate from non‐dilated LV cardiomyopathy to DCM. In our study, patients that progressed to DCM had a higher resting heart rate than those that did not, despite a higher proportion receiving beta‐blockers and similar proportions with a history of atrial fibrillation/flutter. Whilst this subgroup analysis should be considered exploratory due to the small sample size, this finding may indicate that a higher resting heart rate mediates adverse remodelling in early‐NICM. This is in keeping with a post‐hoc analysis of the TRED‐HF trial, which found that a rise in heart rate after withdrawing therapy was associated with relapse and adverse remodelling.^19^

### What this study adds to the literature

We have previously demonstrated the association between fibrosis and SCD in patients with DCM including those whose LVEF had improved to ≥40%;^20^ in this paper we take forward this work by focusing solely on patients with early phenotypic forms of NICM, including patients with intermediate phenotypes (early‐NICM *H+*/*D−* and early‐NICM *H−*/*D+*). There is now emerging data indicating a significant rate of relapse in patients with DCM and improved LVEF, who should be considered as a distinct patient group from early‐NICM.^21^ We have additionally taken forward work from Gigli *et al*.,^22^ who studied 226 patients with early‐DCM or non‐dilated LV cardiomyopathy from an Italian registry demonstrating a low rate of adverse outcomes but identifying non‐sustained VT and restrictive filling on echocardiogram as predictors of worse prognosis. Our study additionally integrates deep phenotyping using CMR, adding important prognostic information and focuses on patients with earlier disease, namely patients with lower symptom burden (71% vs. 42% NYHA class I) and less severe LV dysfunction (median LVEF 55% vs. mean LVEF 34%), compared to the Italian cohort.

### Limitations

The study was from a single centre and its hospital network and inclusion mandated clinical referral for CMR. Whilst this allowed robust and standardized characterization of all patients, we acknowledge the potential for referral bias. Small differences in either LVEDVi or LVEF could have resulted in differential early‐NICM subgroup classification for some patients; we mitigated this through the use of CMR to calculate LV volumes and LVEF, which is the gold standard imaging modality for volumetric analysis and subject to low interobserver variability. We acknowledge that the reference ranges used in this paper for normal LVEDVi and LVEF values were derived from a modest‐sized patient group, but this method offered advantages with respect to the cohort presented in this paper as the reference ranges were calculated using identical analysis technique and software to that presented in this paper and additionally accounted for variation in LVEDVi and LVEF due to age.^9^ We acknowledge the high proportion of Caucasian participants (92%). More regular clinical follow‐up of patients with DCM compared to early‐NICM may have led to disparity in clinical event detection, although we have mitigated this by focusing on hard endpoints. The low event rate and small cohort size may have resulted in limited power to detect differences between early‐NICM subgroups in the risk of clinical outcomes. Due to the cohort size, it was not possible to determine the additive prognostic value of fibrosis characteristics. Further, it is feasible that clinical progression is slow in patients with early‐NICM, as such the median follow‐up duration of 7.9 years may not be enough to demonstrate significant progression in some patients. Obtaining longer follow‐up data for this cohort will be the focus of future work. Two patients experienced MACE due to events that may not have related directly to their cardiomyopathy (one with an acute coronary syndrome, one with a procedural complication). We recognize the subgroup analysis of patients with two CMRs was conducted in those with a clinical referral for a second CMR, introducing the potential for enrichment for those with a change in clinical status. A longer time interval between CMRs and additional data on longitudinal cardiac remodelling through serial echocardiograms in a larger subset of this cohort could provide valuable additional information on phenotypic progression. Genetic testing was not performed systematically in patients in this cohort and cardiac biomarkers were not routinely measured; genetic testing using stored blood samples from this cohort will be the focus of future work.

## Conclusion

Despite only minor morpho‐functional abnormalities and low symptom burden, early‐NICM is not benign. More than one‐quarter of patients with early‐NICM have myocardial fibrosis, which becomes more prominent across the spectrum of NICM severity. Importantly, 11% of patients with early‐NICM experienced MACE over long‐term follow‐up. Identification of patients at risk of SCD remains challenging, but our findings support screening of those at risk and in‐depth investigation when early‐NICM is suspected to aid risk stratification. Further work should focus on whether early targeted therapy reduces the risk of MACE and phenotypic progression in this population.

## Supporting information


**Appendix S1.** Supporting Information.
