# Supplementary material for: Phenotype, outcomes and natural history of early‐stage non‐ischaemic cardiomyopathy
Source: Eur J Heart Fail. 2023 Oct 11;25(11):2050–9. doi: 10.1002/ejhf.3037 (PMC10946699; doi:10.1002/ejhf.3037)
Supplement: Supplementary file 1 — Appendix S1. Supporting Information. [file EJHF-25-2050-s001.docx]

**Supplementary material:**

**Hammersley D.J. *et al.* Phenotype, outcomes and natural history of early-stage non-ischemic cardiomyopathy**

[Supplementary Methods 2](#_Toc136865305)

[Supplementary Figure legends 6](#_Toc136865306)

[Supplementary Tables 8](#_Toc136865307)

[Supplementary Figures 17](#_Toc136865308)

[Supplementary Material references 20](#_Toc136865309)

# Supplementary Methods

*Clinical endpoint definitions*

The following definitions were used by adjudicators for the purpose of adjudicating clinical endpoints in this study.^1,2^

*CV Death: Sudden Cardiac Death*

Death that occurs unexpectedly and not within 30 d of an acute MI. Sudden cardiac death includes the following scenarios:

1. Death witnessed and occurring without new or worsening symptoms.
2. Death witnessed within 60 min of the onset of new or worsening cardiac symptoms unless the symptoms suggest acute MI.
3. Death witnessed and attributed to an identified arrhythmia (e.g., captured on an electrocardiographic recording, witnessed on a monitor, or unwitnessed but found on ICD review).
4. Death after unsuccessful resuscitation from cardiac arrest (e.g., ICD unresponsive sudden cardiac death, pulseless electrical activity arrest).
5. Death after successful resuscitation from cardiac arrest and without identification of a specific cardiac or noncardiac aetiology.
6. Unwitnessed death in a subject seen alive and clinically stable ≤24 h before being found dead without any evidence supporting a specific non-cardiovascular cause of death (information about the patient’s clinical status preceding death should be provided if available)

Unless additional information suggests an alternate specific cause of death (e.g., Death due to Other Cardiovascular Causes), if a patient is seen alive ≤24 h before being found dead, sudden cardiac death (criterion [f]) should be recorded.

*CV Death: Acute MI*

Death by any cardiovascular mechanism (arrhythmia, sudden death, HF, stroke, pulmonary embolus, PAD) within 30 d after an acute MI, related to the immediate consequences of the

MI, such as progressive HF or recalcitrant arrhythmia. There may be assessable (attributable) mechanisms of cardiovascular death during this time period, but for simplicity, if the cardiovascular death occurs within 30 d of an acute MI, it will be considered a death due to MI.

Note: Acute MI should be verified to the extent possible by the diagnostic criteria outlined for acute MI or by autopsy findings showing recent MI or recent coronary thrombosis. Death resulting from a procedure to treat an MI (PCI or CABG), or to treat a complication resulting from MI, should also be considered death due to acute MI. Death resulting from an elective coronary procedure to treat myocardial ischemia (i.e., chronic stable angina) or death due to an MI that occurs as a direct consequence of a cardiovascular investigation / procedure / operation should be considered as a death due to a cardiovascular procedure.

*CV Death: HF*

Death associated with clinically worsening symptoms and/or signs of HF, regardless of HF aetiology.

*CV Death: CVA*

Death after a stroke that is either a direct consequence of the stroke or complication of stroke.

*CV Death: CV Procedure*

Death caused by the immediate complication(s) of a Cardiovascular procedure

*CV Death: CV Haemorrhage*

Death related to haemorrhage such as a non-stroke intracranial haemorrhage, (e.g., subdural hematoma) nonprocedural or nontraumatic vascular rupture (e.g., aortic aneurysm), or haemorrhage causing cardiac tamponade

*CV Death: Other*

Cardiovascular death not included in the above categories but with specific, known cause (e.g., pulmonary embolism).

*Aborted SCD*

Aborted SCD diagnosed if patients have received an appropriate implantable cardioverter-defibrillator (ICD) shock for ventricular arrhythmia, or had a nonfatal episode of ventricular fibrillation or spontaneous sustained ventricular tachycardia causing hemodynamic compromise and requiring cardioversion.

*Sustained VT*

Repetitive ventricular beats in a row lasting over 30 s in duration at a rate greater than 100 beats per minute.

*HF Hospitalisation*

An event where the patient is admitted to the hospital where each of the following criteria apply:

1. Primary diagnosis of HF.
2. Length of stay is at least 24 h (or extends over a calendar date if the hospital admission and discharge times are unavailable).
3. The patient exhibits new or worsening symptoms of HF on presentation and objective evidence of new or worsening HF.
4. Receives initiation or intensification of treatment specifically for HF.

*Unplanned Cardiovascular Hospitalisation*

Where there is an unscheduled admission to hospital due to a cardiovascular diagnosis usually requiring escalation of treatment and often due to some form of decompensation. The admission results in at least a 24-h stay (or a change in calendar date if the hospital admission or discharge times are not available).

# Supplementary Figure Legends

**Supplementary Figure 1: Indexed left ventricular end-diastolic volume and left ventricular ejection fraction calculated from cardiovascular magnetic resonance were used to classify patients with early-stage non-ischaemic cardiomyopathy into phenotypic subgroups, using age- and sex-adjusted reference nomograms values.**

Early-NICM *H-/D+* = isolated left ventricular dilatation; early-NICM *H+/D-* = non-dilated left ventricular cardiomyopathy; early-NICM *H+/D+* = early dilated cardiomyopathy; LVEDVi = indexed left ventricular end-diastolic volume; LVEF = left ventricular ejection fraction

**Supplementary Figure 2: Flow diagram illustrating derivation of the study cohort.** CMR = cardiovascular magnetic resonance; DCM = DCM with left ventricular ejection fraction <50%; early-NICM *H-/D+* = isolated left ventricular dilatation; early-NICM *H+/D-* = non-dilated left ventricular cardiomyopathy; early-NICM *H+/D+* = early dilated cardiomyopathy; HF = heart failure; LVEDVi = indexed left ventricular end-diastolic volume; LVEF = left ventricular ejection fraction; NICM = non-ischemic cardiomyopathy; NYHA = New York Heart Association

**Supplementary Figure 3: Cumulative incidence of life-threatening arrhythmic events stratified by (A) early non-ischemic cardiomyopathy subgroups; and (B) early non-ischemic cardiomyopathy versus dilated cardiomyopathy with LVEF <50%.** There was no difference in the cumulative incidence of life-threatening arrhythmia between early non-ischemic cardiomyopathy subgroups. Patients with dilated cardiomyopathy with left ventricular ejection fraction <50% had a higher cumulative incidence of life-threatening arrhythmia than patients with early non-ischemic cardiomyopathy. DCM = dilated cardiomyopathy with left ventricular ejection fraction <50%; early-NICM = early-stage non-ischemic cardiomyopathy; early-NICM *H-/D+* = isolated left ventricular dilatation; early-NICM *H+/D-* = non-dilated left ventricular cardiomyopathy; early-NICM *H+/D+* = early dilated cardiomyopathy.

**Supplementary Figure 4: Cumulative incidence of major heart failure events stratified by (A) early non-ischemic cardiomyopathy subgroups; and (B) early non-ischemic cardiomyopathy versus dilated cardiomyopathy with LVEF <50%.** There was no difference in the cumulative incidence of major heart failure events between early non-ischemic cardiomyopathy subgroups. Patients with dilated cardiomyopathy with left ventricular ejection fraction <50% had a higher cumulative incidence of major heart failure events than patients with early non-ischemic cardiomyopathy. DCM = dilated cardiomyopathy with left ventricular ejection fraction <50%; early-NICM = early-stage non-ischemic cardiomyopathy; early-NICM *H-/D+* = isolated left ventricular dilatation; early-NICM *H+/D-* = non-dilated left ventricular cardiomyopathy; early-NICM *H+/D+* = early dilated cardiomyopathy.

# Supplementary Tables

**Supplementary Table 1: Comparison of characteristics of patients with early-stage non-ischemic cardiomyopathy and patients with dilated cardiomyopathy with LVEF <50%**

|  | **Early-NICM** | **DCM** | **P** |
| --- | --- | --- | --- |
|  | **n=254** | **n=540** |  |
| **Age, years** | 47 (36-58) | 57 (47-66) | <0.001 |
| **Women** | 94/254 (37) | 172/540 (32) | 0.15 |
| **Caucasian** | 234/254 (92) | 450/540 (83) | <0.001 |
| **Heart rate, bpm** | 70 (60-80) | 76 (65-87) | <0.001 |
| **Systolic blood pressure, mmHg** | 124 (112-138) | 121 (110-134) | 0.04 |
| **Diastolic blood pressure, mmHg** | 73 (65-82) | 73 (64-83) | 0.77 |
| **Body mass index, kg/m^2^** | 26.0 (22.7-29.5) | 26.8 (24.0-30.4) | 0.009 |
| **Diabetes mellitus** | 7/254 (3) | 80/540 (15) | <0.001 |
| **Hypertension** | 60/254 (24) | 168/540 (31) | 0.03 |
| **Current smoker** | 33/252 (13) | 61/537 (11) | 0.28 |
| **History of excess alcohol** | 22/231 (10) | 93/516 (18) | 0.003 |
| **History of chemotherapy** | 6/203 (3) | 29/427 (7) | 0.03 |
| **Peripartum presentation** | 2/235 (1) | 8/507 (2) | 0.34 |
| **History of inherited muscular disease** | 1/207 (1) | 3/437 (1) | 0.61 |
| **Family history of dilated cardiomyopathy** | 85/211 (40) | 57/470 (12) | <0.001 |
| **Family history of SCD** | 45/202 (22) | 64/438 (15) | 0.02 |
| **Atrial fibrillation/flutter** | 44/228 (19) | 129/459 (28) | 0.01 |
| **Left bundle branch block** | 29/254 (11) | 160/540 (30) | <0.001 |
| **Non-sustained VT** | 21/176 (12) | 66/229 (29) | <0.001 |
| **NYHA** |  |  | <0.001 |
| 1 | 181/254 (71) | 215/539 (40) |  |
| 2 | 73/254 (29) | 232/539 (43) |  |
| 3 | 0/254 (0) | 84/539 (16) |  |
| 4 | 0/254 (0) | 8/539 (1) |  |
| **ACE-i or ARB** | 132/253 (52) | 472/540 (87) | <0.001 |
| **Beta-blocker** | 97/253 (38) | 423/540 (78) | <0.001 |
| **Mineralocorticoid receptor antagonist** | 20/253 (8) | 240/538 (45) | <0.001 |
| Data presented as median (IQR) or n/N (%). ACEi = angiotensin-converting enzyme inhibitor; ARB = angiotensin II receptor blocker; bpm = beats per minute; DCM = dilated cardiomyopathy with left ventricular ejection fraction <50%; Early-NICM = early non-ischemic cardiomyopathy; kg = kilogram; m = meter; NYHA = New York Heart Association; SCD = sudden cardiac death; VT = ventricular tachycardia. | | | |

**Supplementary Table 2: Phenotype of patients with early-non ischemic cardiomyopathy compared to patients with dilated cardiomyopathy and LVEF <50%**

| ***All patients with early-NICM and DCM*** | | | |
| --- | --- | --- | --- |
|  |  |  |  |
|  | **Early-NICM** | **DCM** |  |
|  | **n=254** | **n=540** | **P** |
| **LVEDVi, ml/m^2^** | 101 (90-108) | 127 (109-153) | <0.001 |
| **LVESVi, ml/m^2^** | 44 (40-50) | 82 (65-108) | <0.001 |
| **LVEF, %** | 55 (52-59) | 34 (26-43) | <0.001 |
| **Indexed LV mass, g/m^2^** | 74 (64-83) | 89 (76-110) | <0.001 |
| **RVEDVi, ml/m^2^** | 91 (78-106) | 83 (68-101) | 0.003 |
| **RVESVi, ml/m^2^** | 39 (31-47) | 41 (29-58) | 0.08 |
| **RVEF, %** | 57 (52-63) | 50 (39-60) | <0.001 |
| **LAVi, ml/m^2^** | 51 (42-61) | 57 (47-75) | <0.001 |
| **Maximum LV wall thickness, mm** | 10.0 (8.0-11.0) | 10.0 (8.0-11.0) | 0.33 |
| **Mean septal thickness, mm** | 7.5 (6.0-9.0) | 8.0 (6.5-9.0) | 0.08 |
| **Mean lateral wall thickness, mm** | 5.5 (4.0-6.5) | 5.5 (4.5-7.0) | 0.11 |
| **Myocardial fibrosis present (%)** | 65/254 (26) | 207/533 (39) | <0.001 |
| **Fibrosis position (% of patients)** |  |  |  |
| Septal | 45/254 (18) | 182/533 (34) | <0.001 |
| Free-wall | 34/254 (13) | 93/533 (17) | 0.18 |
| **Fibrosis pattern (% of patients)** |  |  |  |
| Mid-wall | 59/254 (23) | 195/533 (36) | <0.001 |
| Subepicardial | 18/254 (7) | 37/533 (7) | 0.88 |
| ***Patients with early-NICM and DCM with myocardial fibrosis*** | | | |
|  | **Early-NICM**  **n=65** | **DCM**  **n=207** | **P** |
| **Fibrosis mass (5SD method)** |  |  |  |
| Absolute mass, g | 1.6 (0.9-2.7) | 2.2 (1.3-3.6) | 0.007 |
| Relative mass, % | 1.2 (0.7-2.1) | 1.5 (0.8-2.4) | 0.52 |
| Data presented as median (IQR) or n/N (%). 5SD = 5-standard deviation; DCM = dilated cardiomyopathy with left ventricular ejection fraction <50%; Early-NICM = early non-ischemic cardiomyopathy; g = grams; LAVi = left atrial volume index; LV = left ventricular; LVEDVi = left ventricular end-diastolic volume index; LVEF = left ventricular ejection fraction; LVESVi = left ventricular end-systolic volume index; m = meter; mm = millimeter; ml = milliliter; RVEDVi = right ventricular end-diastolic volume index; RVEF = right ventricular ejection fraction; RVESVi = right ventricular end-systolic volume index. | | | |

**Supplementary Table 3: Comparison of early-stage non-ischaemic cardiomyopathy patients that did and did not experience major adverse cardiovascular events during follow-up**

|  | **Early-NICM - no MACE** | **Early-NICM - MACE** | **P** |
| --- | --- | --- | --- |
|  | **n=226** | **n=28** |  |
| **Age, years** | 46 (35-57) | 55 (45-67) | 0.002 |
| **Female** | 82 (36) | 12 (43) | 0.50 |
| **Caucasian** | 211/226 (93) | 23/28 (82) | 0.04 |
| **Heart rate, bpm** | 70 (61-81) | 66 (58-77) | 0.26 |
| **Systolic blood pressure, mmHg** | 123 (112-137) | 127 (114-139) | 0.47 |
| **Diastolic blood pressure, mmHg** | 72 (65-82) | 73 (68-82) | 0.80 |
| **Body mass index, kg/m2** | 25.9 (22.6-29.2) | 27.9 (24.8-32.6) | 0.01 |
| **Diabetes mellitus** | 3/226 (1) | 4/28 (14) | 0.003 |
| **Hypertension** | 52/226 (23) | 8/28 (29) | 0.51 |
| **Current smoker** | 29/225 (13) | 4/27 (15) | 0.48 |
| **History of excess alcohol** | 19/206 (9) | 3/25 (12) | 0.43 |
| **History of chemotherapy** | 6/180 (3) | 0/23 (0) | 0.48 |
| **Peripartum presentation** | 1/210 (0) | 1/25 (4) | 0.20 |
| **History of inherited muscular disease** | 1/185 (1) | 0/22 (0) | 0.89 |
| **Family history of DCM** | 82/186 (44) | 3/25 (12) | 0.001 |
| **Family history of SCD** | 41/181 (23) | 4/21 (19) | 0.48 |
| **Atrial fibrillation/flutter** | 36/202 (18) | 8/26 (31) | 0.12 |
| **Left bundle branch block** | 23/226 (10) | 6/28 (21) | 0.08 |
| **Non-sustained VT** | 12/156 (8) | 9/20 (45) | <0.001 |
| **NYHA** |  |  | 0.64 |
| 1 | 160/226 (71) | 21/28 (75) |  |
| 2 | 66/226 (29) | 7/28 (25) |  |
| **ACE-i or ARB** | 109/225 (48) | 23/28 (82) | <0.001 |
| **Beta-blocker** | 80/225 (36) | 17/28 (61) | 0.010 |
| **Mineralocorticoid receptor antagonist** | 13/225 (6) | 7/28 (25) | <0.001 |
| **LVEDVi, ml/m2** | 101 (90-108) | 104 (90-118) | 0.38 |
| **LVESVi, ml/m2** | 43 (39-49) | 48 (43-54) | 0.03 |
| **LVEF, %** | 55 (52-59) | 54 (52-56) | 0.14 |
| **Indexed LV mass, g/m2** | 74 (63-82) | 78 (65-101) | 0.09 |
| **RVEDVi, ml/m2** | 92 (78-106) | 87 (80-104) | 0.62 |
| **RVESVi, ml/m2** | 39 (31-47) | 41 (31-48) | 0.74 |
| **RVEF, %** | 57 (52-63) | 57 (48-61) | 0.50 |
| **LAVi, ml/m2** | 51 (42-60) | 60 (45-76) | 0.02 |
| **Mid-wall fibrosis present** | 53/226 (23) | 12/28 (46) | 0.01 |
| Data presented as median (IQR) or n/N (%). ACEi = angiotensin-converting enzyme inhibitor; ARB = angiotensin II receptor blocker; bpm = beats per minute; CMR = cardiovascular magnetic resonance; DCM = dilated cardiomyopathy; early-NICM = early non-ischaemic cardiomyopathy; kg = kilogram; LAVi = left atrial volume index; LV = left ventricular; LVEDVi = left ventricular end-diastolic volume index; LVEF = left ventricular ejection fraction; LVESVi = left ventricular end-systolic volume index; m = meter; NYHA = New York Heart Association; RVEDVi = right ventricular end-diastolic volume index; RVEF = right ventricular ejection fraction; RVESVi = right ventricular end-systolic volume index; SCD = sudden cardiac death; VT = ventricular tachycardia. | | | |

**Supplementary Table 4: Univariate and multivariable associations of patient and cardiovascular magnetic resonance characteristics with MACE in patients with early non-ischemic cardiomyopathy**

|  | **Univariable** | | | **Multivariable** | | |
| --- | --- | --- | --- | --- | --- | --- |
| **Variable** | **HR** | **95% CI** | **P** | **HR** | **95% CI** | **P** |
| **Age (per 10 years)** | 1.54 | 1.16 - 2.04 | 0.003 |  |  |  |
| **Female sex** | 1.27 | 0.60 - 2.68 | 0.54 |  |  |  |
| **Caucasian** | 0.32 | 0.12 - 0.84 | 0.02 |  |  |  |
| **Heart rate (per 10bpm)** | 0.88 | 0.67 - 1.16 | 0.37 |  |  |  |
| **Systolic blood pressure (per 10mmHg)** | 1.10 | 0.89 - 1.36 | 0.38 |  |  |  |
| **Diastolic blood pressure (per 10mmHg)** | 0.98 | 0.71 - 1.35 | 0.89 |  |  |  |
| **Body mass index, kg/m^2^** | 1.07 | 1.01 - 1.14 | 0.02 |  |  |  |
| **Diabetes mellitus** | 6.72 | 2.33 - 19.41 | <0.001 | 5.12 | 1.73 - 15.18 | 0.003 |
| **Hypertension** | 1.25 | 0.55 - 2.85 | 0.59 |  |  |  |
| **Current smoker** | 0.97 | 0.34 - 2.80 | 0.96 |  |  |  |
| **History of excess alcohol** | 0.95 | 0.29 - 3.16 | 0.94 |  |  |  |
| **History of chemotherapy** | 0.99 | 0.13 - 7.31 | 0.99 |  |  |  |
| **Peripartum presentation** | 5.45 | 1.29 - 23.06 | 0.02 |  |  |  |
| **History of inherited muscular disease** | 0.00 | NA | 1.00 |  |  |  |
| **Family history of DCM** | 0.18 | 0.06 - 0.60 | 0.005 | 0.18 | 0.05 - 0.59 | 0.005 |
| **Family history of SCD** | 0.49 | 0.17 - 1.41 | 0.18 |  | | |
| **Atrial fibrillation/flutter** | 1.76 | 0.79 - 3.89 | 0.16 |  | | |
| **Left bundle branch block** | 2.07 | 0.84 - 5.11 | 0.11 |  |  |  |
| **Non-sustained VT** | 3.84 | 1.80 - 8.19 | <0.001 | 5.10 | 2.36 - 11.00 | <0.001 |
| **NYHA** |  |  | 0.68 |  | | |
| 1 | Reference |  |  |  |  |  |
| 2 | 0.84 | 0.36 - 1.97 |  |  |  |  |
| **CMR indication** |  |  | 0.003 |  |  |  |
| Characterisation LV dysfunction/dilation | Reference |  |  |  |  |  |
| Arrhythmia | 2.73 | 1.18 - 6.32 |  |  |  |  |
| Family screen | 0.13 | 0.02 - 1.02 |  |  |  |  |
| Other | 0.89 | 0.27 - 2.89 |  |  |  |  |
| **ACE-i or ARB** | 4.10 | 1.56 - 10.79 | 0.004 |  |  |  |
| **Beta-blocker** | 2.33 | 1.09 - 4.98 | 0.03 |  |  |  |
| **Mineralocorticoid receptor antagonist** | 4.79 | 2.02 - 11.35 | <0.001 |  |  |  |
| **LVEDV (log), ml** | 4.38 | 0.64 - 30.24 | 0.13 |  |  |  |
| **LVEDVi (log), ml/m^2^** | 3.67 | 0.39 - 34.88 | 0.26 |  |  |  |
| **LVESV (log), ml** | 8.57 | 1.40 - 52.51 | 0.02 |  |  |  |
| **LVESVi (log), ml/m^2^** | 8.06 | 1.10 - 58.97 | 0.04 |  |  |  |
| **LVEF, %** | 0.97 | 0.92 - 1.02 | 0.21 |  |  |  |
| **LV mass (log), g** | 3.12 | 0.86 - 11.22 | 0.08 |  |  |  |
| **Indexed LV mass (log), g/m^2^** | 3.55 | 0.78 - 16.16 | 0.10 |  |  |  |
| **RVEDV (log), ml** | 1.06 | 0.26 - 4.31 | 0.94 |  |  |  |
| **RVEDVi (log), ml/m^2^** | 0.74 | 0.15, 3.67 | 0.71 |  |  |  |
| **RVESV (log), ml** | 1.44 | 0.50 - 4.16 | 0.50 |  |  |  |
| **RVESVi (log), ml/m^2^** | 1.33 | 0.41 - 4.27 | 0.63 |  |  |  |
| **RVEF, %** | 0.98 | 0.94 - 1.02 | 0.34 |  |  |  |
| **LAVi, ml/m^2^** | 6.20 | 1.67 - 23.05 | 0.006 |  |  |  |
| **Maximum LV wall thickness, mm** | 1.04 | 0.87 - 1.24 | 0.69 |  |  |  |
| **Mean septal thickness, mm** | 1.06 | 0.87 - 1.30 | 0.54 |  |  |  |
| **Mean lateral wall thickness, mm** | 0.96 | 0.74 - 1.24 | 0.75 |  |  |  |
| **Mid-wall fibrosis present** | 2.84 | 1.35 - 5.97 | 0.006 | 3.77 | 1.73 - 8.20 | <0.001 |
| Data presented as univariable and multivariable hazard ratios with 95% confidence intervals. ACEi = angiotensin-converting enzyme inhibitor; ARB = angiotensin II receptor blocker; CI = confidence interval; CMR = cardiovascular magnetic resonance; DCM = dilated cardiomyopathy; g = grams; HR = hazard ratio; kg = kilogram; LAVi = left atrial volume index; LV = left ventricular; LVEDV = left ventricular end-diastolic volume; LVEDVi = left ventricular end-diastolic volume index; LVEF = left ventricular ejection fraction; LVESV = left ventricular end-systolic volume; LVESVi = left ventricular end-systolic volume index; m = meter; mm = millimeter; ml = milliliter; RVEDV = right ventricular end-diastolic volume; RVEDVi = right ventricular end-diastolic volume index; RVEF = right ventricular ejection fraction; RVESV = right ventricular end-systolic volume; RVESVi = right ventricular end-systolic volume index; SCD = sudden cardiac death; VT = ventricular tachycardia. | | | | | | |

**Supplementary Table 5: Incidence rate per 100 patients-years for secondary clinical endpoints in patients with early non-ischemic cardiomyopathy and dilated cardiomyopathy with LVEF <50%**

|  | **Early-NICM** | | **DCM** | |  |
| --- | --- | --- | --- | --- | --- |
|  | **No. events** | **Incidence rate per 100 patient-years (95% CI)** | **No. events** | **Incidence rate per 100 patient-years (95% CI)** | **Rate ratio (95% CI)** |
| **Life-threatening arrhythmia** | 11 | 0.54 (0.30-0.98) | 39 | 1.02 (0.74-1.39) | 1.87  (0.94-4.05) |
| **Major heart failure events** | 9 | 0.44 (0.23-0.85) | 107 | 2.90 (2.40-3.50) | 6.54  (3.32-14.71) |
| Data presented as event numbers and incidence rates per 100 patient-years with 95% confidence intervals and rate ratios. CI = confidence interval; DCM = dilated cardiomyopathy with left ventricular ejection fraction <50%); Early-NICM = early non-ischemic cardiomyopathy. | | | | | |

**Supplementary Table 6: Comparison of baseline characteristics of patients that did and did not progress from early non-ischemic cardiomyopathy to dilated cardiomyopathy with LVEF <50%**

|  | **Progression to DCM on follow-up CMR** | |  |
| --- | --- | --- | --- |
| **Baseline characteristics** | **No** | **Yes** | **P** |
|  | **n=110** | **n=9** |  |
| **Age, years** | 43 (32-54) | 41 (31-54) | 0.89 |
| **Women** | 45/110 (41) | 1/9 (11) | 0.08 |
| **Caucasian** | 102/110 (93) | 7/9 (78) | 0.12 |
| **Heart rate, bpm** | 70 (61-77) | 89 (72-95) | <0.001 |
| **Systolic blood pressure, mmHg** | 120 (112-133) | 123 (116-129) | 0.21 |
| **Diastolic blood pressure, mmHg** | 71 (64-80) | 73 (70-76) | 0.87 |
| **Body mass index, kg/m^2^** | 25.8 (22.0-29.4) | 24.4 (22.1-27.3) | 0.43 |
| **Diabetes mellitus** | 2/110 (2) | 0/9 (0) | 0.68 |
| **Hypertension** | 18/110 (16) | 1/9 (11) | 0.68 |
| **Current smoker** | 11/109 (10) | 5/9 (56) | 0.002 |
| **History of excess alcohol** | 6/105 (6) | 2/8 (25) | 0.04 |
| **Family history of DCM** | 53/97 (55) | 3/8 (38) | 0.35 |
| **Family history of SCD** | 23/93 (25) | 1/7 (14) | 0.53 |
| **Atrial fibrillation/flutter** | 14/101 (14) | 0/9 (0) | 0.23 |
| **Left bundle branch block** | 3/110 (3) | 4/9 (44) | <0.001 |
| **Non-sustained VT** | 9/86 (10) | 0/8 (0) | 0.34 |
| **NYHA** |  |  | 0.71 |
| 1 | 91/110 (83) | 7/9 (78) |  |
| 2 | 19/110 (17) | 2/9 (22) |  |
| **ACE-i or ARB** | 43/110 (39) | 6/9 (67) | 0.11 |
| **Beta-blocker** | 29/110 (26) | 7/9 (78) | 0.001 |
| **Mineralocorticoid receptor antagonist** | 4/110 (4) | 1/9 (11) | 0.28 |
| Data presented as median (IQR) or n/N (%). ACEi = angiotensin-converting enzyme inhibitor; ARB = angiotensin II receptor blocker; bpm = beats per minute; DCM = dilated cardiomyopathy; DCM = dilated cardiomyopathy with left ventricular ejection fraction <50%; kg = kilogram; NYHA = New York Heart Association; SCD = sudden cardiac death; VT = ventricular tachycardia. | | | |

**Supplementary Table 7: Comparison of baseline CMR characteristics of patients that did and did not progress from early non-ischemic cardiomyopathy to dilated cardiomyopathy with LVEF <50% on follow up CMR**

|  | **Progression to DCM on follow-up CMR** | |  |
| --- | --- | --- | --- |
| **CMR characteristics** | **No** | **Yes** | **P** |
|  | **n=110** | **n=9** |  |
| **LVEDVi, ml/m^2^** | 102 (90-108) | 100 (99-109) | 0.75 |
| **LVESVi, ml/m^2^** | 43 (38-47) | 50 (43-52) | 0.04 |
| **LVEF, %** | 57 (54-59) | 54 (52-55) | 0.02 |
| **Indexed LV mass, g/m^2^** | 72 (61-82) | 81 (75-84) | 0.31 |
| **RVEDVi, ml/m^2^** | 94 (82-106) | 87 (76-96) | 0.17 |
| **RVESVi, ml/m^2^** | 39 (31-46) | 39 (26-41) | 0.31 |
| **RVEF, %** | 57 (54-64) | 58 (54-65) | 0.71 |
| **LAVi, ml/m^2^** | 50 (40-60) | 43 (37-57) | 0.35 |
| **Maximum LV wall thickness, mm** | 9.0 (8.0-11.0) | 9.5 (8.0-10.0) | 0.77 |
| **Mean septal thickness, mm** | 7.0 (6.0-8.0) | 8.5 (7.5-8.5) | 0.22 |
| **Mean lateral wall thickness, mm** | 5.0 (4.0-6.0) | 5.5 (5.0-7.0) | 0.29 |
| **Mid-wall fibrosis present** | 24/110 (22) | 2/9 (22) | 1.00 |
| Data presented as median (IQR) or n/N (%). DCM = dilated cardiomyopathy with left ventricular ejection fraction <50%; g = grams; LAVi = left atrial volume index; LV = left ventricular; LVEDVi = left ventricular end-diastolic volume index; LVEF = left ventricular ejection fraction; LVESVi = left ventricular end-systolic volume index; m = meter; mm = millimeter; ml = milliliter; RVEDVi = right ventricular end-diastolic volume index; RVEF = right ventricular ejection fraction; RVESVi = right ventricular end-systolic volume index. | | | |

# Supplementary Figures

**Supplementary Figure 1**

**
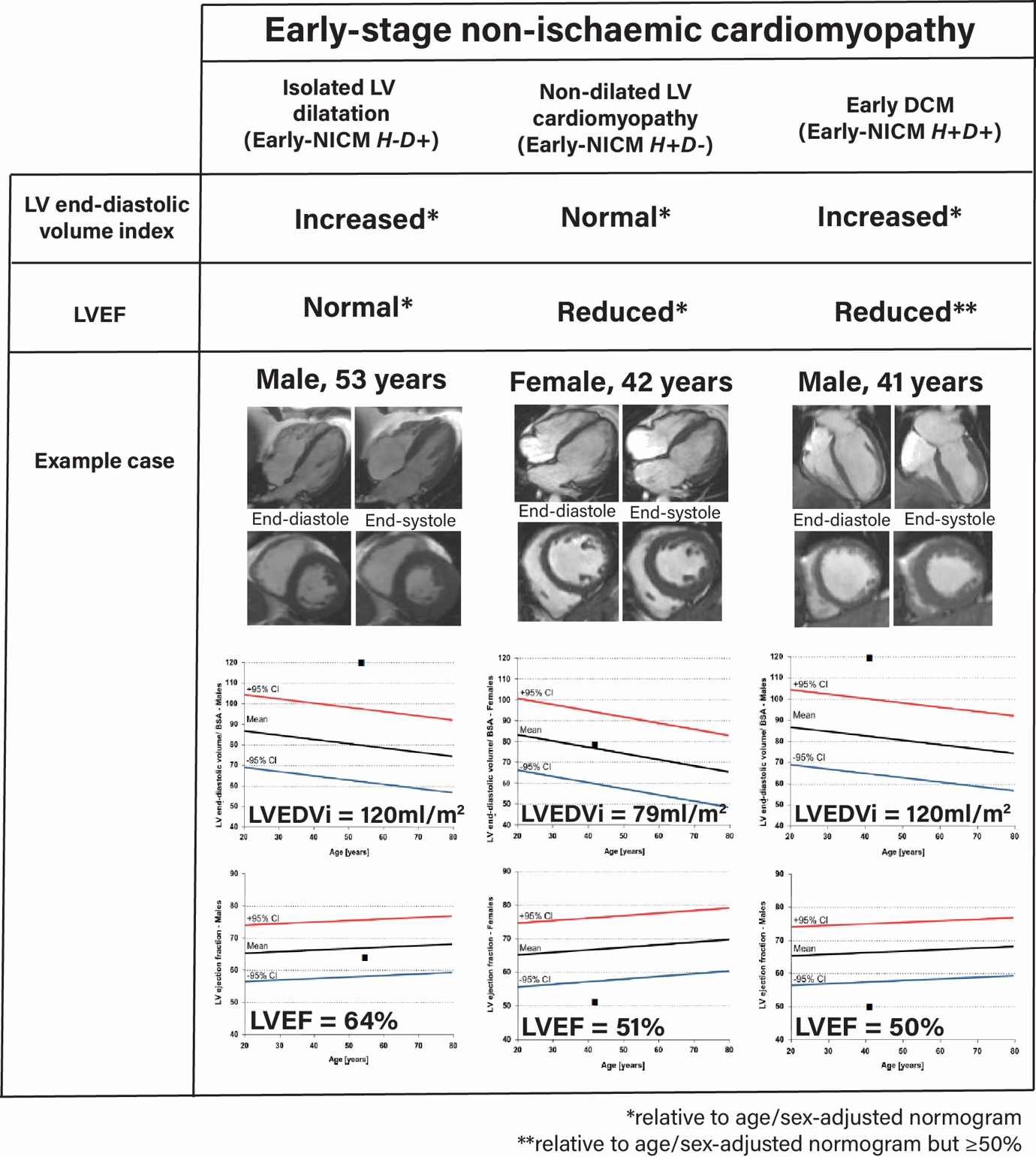
**

**Supplementary Figure 2**


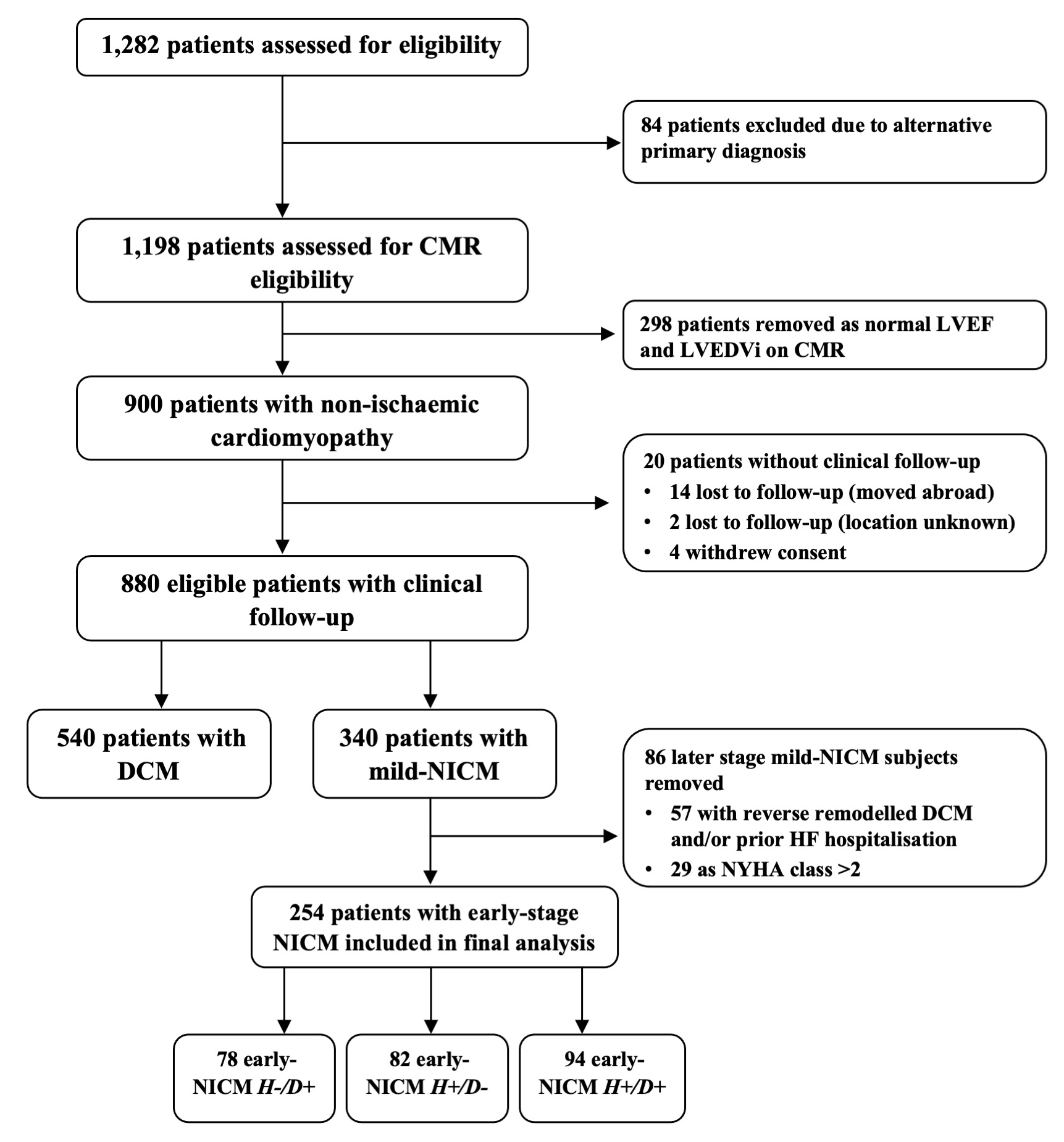


**Supplementary Figure 3**


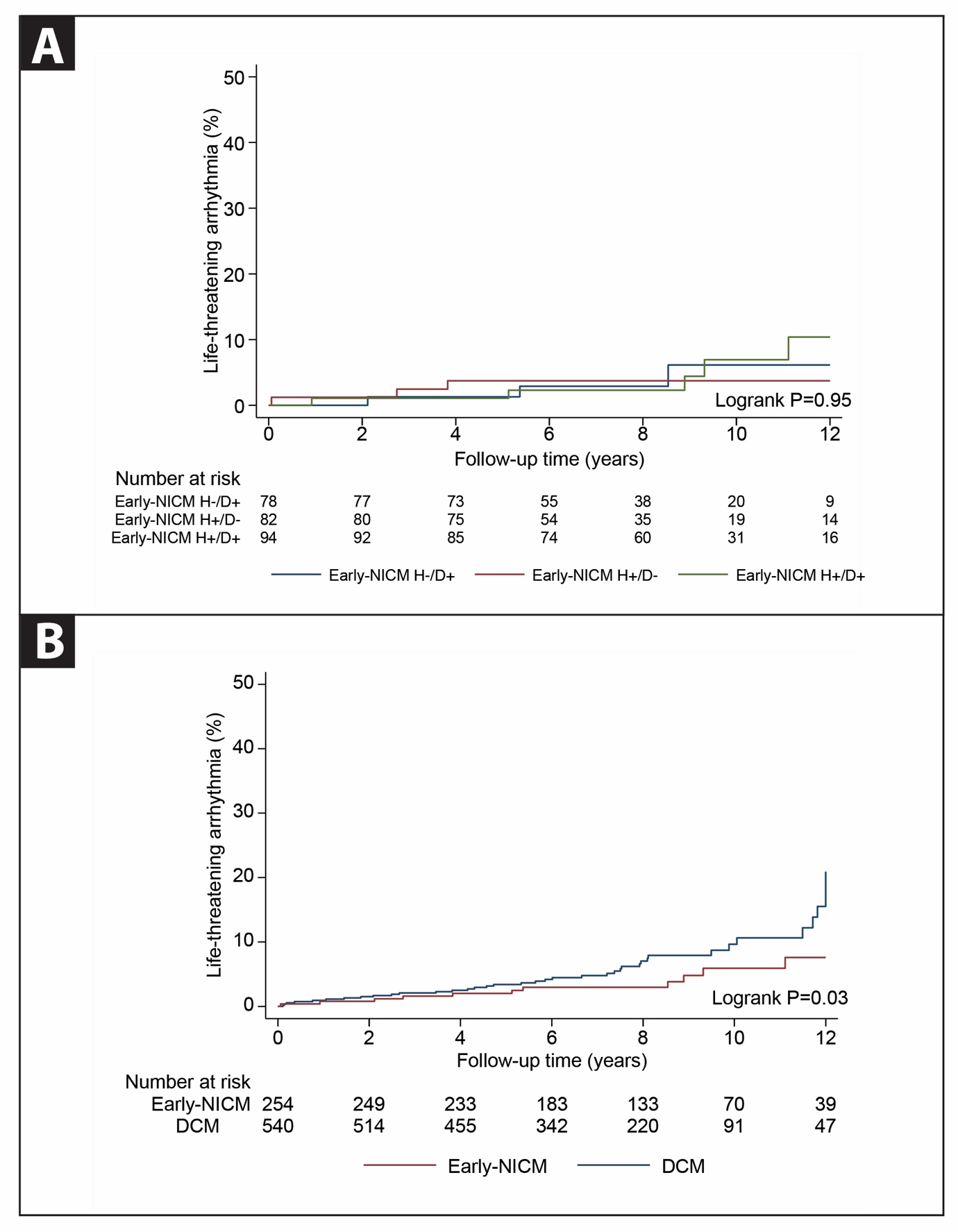


**Supplementary Figure 4**

**
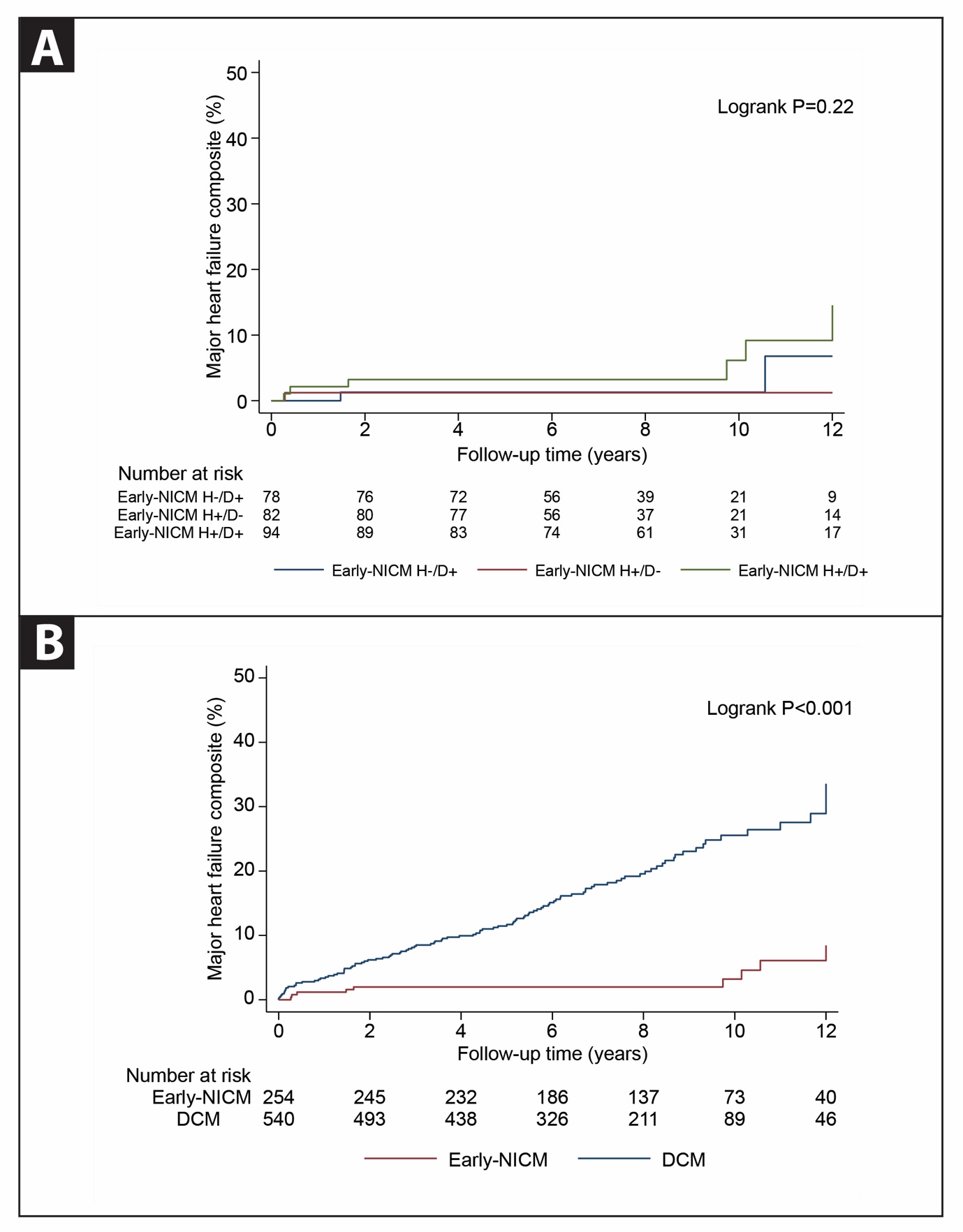
**

# Supplementary Material References

1. Hicks KA, Tcheng JE, Bozkurt B, Chaitman BR, Cutlip DE, Farb A, Fonarow GC, Jacobs JP, Jaff MR, Lichtman JH, Limacher MC, Mahaffey KW, Mehran R, Nissen SE, Smith EE, Targum SL. 2014 ACC/AHA key data elements and definitions for cardiovascular endpoint events in clinical trials: A Report of the American College of Cardiology/American Heart Association Task Force on Clinical Data Standards (Writing Committee to Develop Cardiovascu. *J Am Coll Cardiol* 2015;**66**:403–469.

2. American College of Cardiology/American Heart Association Task Force on Clinical Data Standards (ACC/AHA/HRS Writing Committee to Develop Data Standards on Electrophysiology), Buxton AE, Calkins H, Callans DJ, DiMarco JP, Fisher JD, Greene HL, Haines DE, Hayes DL, Heidenreich PA, Miller JM, Poppas A, Prystowsky EN, Schoenfeld MH, Zimetbaum PJ, Goff DC, Grover FL, Malenka DJ, Peterson ED, Radford MJ, Redberg RF. ACC/AHA/HRS 2006 key data elements and definitions for electrophysiological studies and procedures: a report of the American College of Cardiology/American Heart Association Task Force on Clinical Data Standards (ACC/AHA/HRS Writing Committee to Develop D. *Circulation* 2006;**114**:2534–2570.
